# Familial Associations in Testicular Cancer with Other Cancers

**DOI:** 10.1038/s41598-018-28819-7

**Published:** 2018-07-18

**Authors:** Luyao Zhang, Hongyao Yu, Otto Hemminki, Asta Försti, Kristina Sundquist, Kari Hemminki

**Affiliations:** 1 Division of Molecular Genetic Epidemiology, German Cancer Research Center (DKFZ), Im Neuenheimer Feld 580, D-69120 Heidelberg, Germany; 2 Department of Abdominal Surgery and Urology, Helsinki University Hospital, Helsinki, Finland; 3 Cancer Gene Therapy Group, Faculty of Medicine, University of Helsinki, Helsinki, Finland; 4 Center for Primary Health Care Research, Lund University, 205 02 Malmö, Sweden; 5 Department of Family Medicine and Community Health, Department of Population Health Science and Policy, Icahn School of Medicine at Mount Sinai, New York, USA; 6 Center for Community-based Healthcare Research and Education (CoHRE), Department of Functional Pathology, School of Medicine, Shimane University, Shimane, Japan

## Abstract

Familial risks for testicular cancer (TC) are among the highest of all cancers. However, data are limited for histological types of TC and for possible familial associations of TC with other cancers. We used the nationwide Swedish Family-Cancer Database for years 1958 to 2015 to analyse familial relative risks (RR) for 11,138 TC patients when first-degree relatives were diagnosed with TC or other cancer in reference to those without a family history. A total of 191 familial TCs were found, which accounted for 2.0% of all TC. The RR was 5.06 when one family member was diagnosed with TC with no significant difference between seminoma and nonseminoma. However, the risk for nonseminoma was 33.59 when two family members were affected. Internally consistent familial associations of TC, particularly of seminoma, were found with breast and nervous system cancers and melanoma. Individual significant associations were found for a number of sites, including ovarian, endometrial and prostate cancers. Our results suggest that nonseminoma may have a stronger genetic background than seminoma but seminoma shares more familial associations with discordant cancers. Clustering of TC with hormone-dependent cancers of the breast, ovary, endometrium and prostate may suggest mechanistic links and possibly gene-environment interactions.

## Introduction

The incidence of testicular cancer (TC) varies with ethnic origin and with economic state, the incidence being highest in developed countries and lowest in developing countries^1^. In Europe, the incidence of TC has dramatically increased, concomitantly with an equally dramatic decrease in mortality^2,3^. Differences in incidence between the countries have been among the largest for any cancer. The increasing trend has been described as a birth cohort effect but in the high-incidence Scandinavian countries the increase has leveled off^2,4^. Precursor lesions of TC are thought to arise and accumulate as a result of aberrant fetal gonocyte development and these *in-situ* lesions gain invasive phenotype in early adulthood^5,6^. Individual risk factors of TC include cryptorchidism, hypospadias, inguinal hernia, subfertility and other lower risk birth-related factors^7,8^. Some of these factors are understood to be related to androgen insufficiency^6^. Environmental pollutants have been associated with the risk of TC; e.g. the type of organochlorine compounds among endocrine disruptive chemicals^6,8^. The role of environmental factors was documented among low-risk Finnish immigrants to Sweden whose TC incidence was only 0.45 of the Swedish rate^9^. The risk of TC in Sweden-born sons of Finnish immigrant parents was no longer different from native Swedes, which implies a strong environmental influence in spite of the 100% Finnish genotype^9^. In a family study on cancer etiology, TC was found to be the cancer with the highest proportion of childhood shared environmental effects among all assessed causes^10^. These results combined suggest that environmental factors during childhood and adolescence affect TC risk^6,11^. Finding these factors and their possible interactions with host genetic background would likely help to resolve the issue of increasing incidence trends.

Familial risk of TC is among the highest of all cancers but the risk is significantly higher if the affected family member is a brother (6.94) rather than a father (3.90), which is likely related to birth cohort effects^12^. Approximately 1.8% of TC patients have a father or a sibling diagnosed with TC^12^. According to a Nordic TC study, the risk of seminoma in brothers (SIR 4.2) was not different from the risk of all TC in brothers (4.1)^13^. TC has been shown to be associated with other (discordant) cancers in families including lung, kidney and oesophageal cancers, melanoma, leukaemia and non-Hodgkin lymphomas^14,15^. Our previous study covered the years 1958 to 2002 and used data from the Swedish Family-Cancer Database^14^. As we have recently updated the database with cancers up to the year 2015, we decided to revisit, here in this study, familial associations of 11,138 TCs with TC and discordant cancers.

## Results

We identified a total of 11,138 TC of which 9711 were identified in offspring generations used in the RR calculations. Seminomas (6120) outnumbered nonseminomas (4729) in the total population and among the offspring generations (5133 and 4391). The median diagnostic ages for seminoma were higher (38 years) than for nonseminoma (29 years) for all, and in the offspring generations these were 36 and 28 years respectively. All cancers in the database amounted to 1.96 million, of which 701,617 were diagnosed in the offspring generations.

Table 1 shows familial risk for TC in sons when a first-degree family member was also diagnosed with TC. A total of 191 familial TCs were found in the offspring generation, which accounted for 2.0% of all TC (191/9711). RR was 5.06 when one proband was diagnosed with TC and it was 18.23 when two probands were affected. RR was 5.55 for seminoma and 4.46 for nonseminoma when one proband was diagnosed with TC. The risk for nonseminoma was 33.59 when two probands were diagnosed with TC; however these were only 4 cases. The results were essentially similar in the reverse order analysis: risk for TC in sons when family members were diagnosed with seminoma or nonseminoma (lower two lines in Table 1). The risk of TC was 96.78 when two family members were diagnosed with nonseminoma.

**Table 1:** Familial risks for histology-specific testicular cancer (TC).

Testicular cancer histology	Cases with negative family history	Cases with 1 FDR			Cases with 2 FDRs		
		Cases	RR	95%CI	Cases	RR	95%CI
**Histology in sons**							
Seminoma	5024	108	**5**.**55**	4.59–6.72	1	6.69	0.94–47.52
Non-seminoma	4313	74	**4**.**46**	3.54–5.61	4	**33**.**59**	12.60–89.56
**Histology in relatives**							
Seminoma	9606	104	**4**.**96**	4.09–6.02	1	**11**.**91**	1.68–84.58
Non-seminoma	9630	77	**4**.**92**	3.93–6.15	4	**96**.**78**	36.31–257.94
All testicular cancer	9520	186	**5**.**06**	4.38–5.86	5	**18**.**23**	7.59–43.81

Bolding of RRs shows that 95%CI does not include 1.00.

FDRs = first degree relatives. RR = relative risk. CI = confidence interval.

Table 2 shows the risk for TC in sons depending on cancers in their family members, and in reverse order, the risk of cancer in the offspring generation when family members were diagnosed with testicular cancers. At least 20 testicular cancers had to be recorded with any cancer in relatives for the site to be listed. The RR for TC was increased when family members were diagnosed with lung (1.11), breast (1.09) and nervous system (1.17) cancer and with melanoma (1.24). TC risk was increased to 1.12 when family members were diagnosed with any cancer. In the reverse order, risk for any cancer in offspring when family members were diagnosed with TC, the results remained significant for breast cancer and melanoma while pancreatic and nervous system cancers lost significance. However, a new association of prostate cancer with TC (RR 1.15) emerged. We also carried out an analysis of familial risk according to the number of cancers diagnosed in a family, similar to Table 1 (data not shown). The only significant association was the RR for ovarian cancer in families of two TC patients (3 families, RR 3.49, 95%CI 1.13–10.84).

**Table 2:** Risk of testicular cancer in sons when family members were diagnosed with any cancer.

Cancer site family member	Cases with negative family history	Relatives with discordant cancers			Cases with negative family history	Relatives with testicular cancers		
		Any FDRs				Any FDRs		
		Cases	RR	95%CI		Cases	RR	95%CI
Upper aerodigestive tract	9620	91	0.84	0.68–1.03	14604	49	0.85	0.64–1.12
Stomach	9605	106	0.76	0.63–0.92	10633	29	0.69	0.48–1.00
Colorectum	9157	554	1.06	0.97–1.15	61007	246	1.00	0.88–1.13
Liver	9620	91	0.81	0.66–1.00	11383	40	0.90	0.66–1.23
Pancreas	9575	136	1.16	0.98–1.37	12414	63	1.27	0.99–1.63
Lung	9312	399	**1**.**11**	1.00–1.23	45724	187	1.04	0.90–1.21
Breast (female and male)	8955	756	**1**.**09**	1.01–1.17	117258	544	**1**.**09**	1.00–1.18
Cervix	9619	92	0.96	0.79–1.18	13355	52	0.94	0.71–1.23
Endometrium	9565	146	1.09	0.92–1.28	18086	67	0.89	0.70–1.14
Ovary	9590	121	1.12	0.94–1.34	15388	67	1.03	0.81–1.31
Prostate	8877	834	1.03	0.96–1.11	104329	497	**1**.**15**	1.05–1.25
Kidney	9593	118	0.86	0.72–1.03	16093	62	0.96	0.75–1.23
Urinary bladder	9475	236	1.05	0.92–1.19	24819	101	1.03	0.85–1.25
Melanoma	9426	285	**1**.**24**	1.10–1.40	41537	201	**1**.**16**	1.01–1.33
Skin	9524	187	0.87	0.77–1.02	21917	85	0.98	0.79–1.21
Nervous system	9497	214	**1.17**	1.02–1.34	32146	140	1.07	0.90–1.26
Thyroid glands	9658	53	1.08	0.83–1.42	8640	36	1.02	0.73–1.41
Endocrine glands	9623	88	0.91	0.74–1.12	14964	53	0.85	0.65–1.12
Connective tissue	9673	38	1.07	0.78–1.47	5549	22	0.98	0.65–1.49
Non-Hodgkin lymphoma	9546	165	1.01	0.87–1.18	23160	103	1.10	0.91–1.34
Hodgkin lymphoma	9682	29	0.93	0.64–1.34	6185	17	0.68	0.42–1.10
Myeloma	9650	61	0.93	0.72–1.20	7031	27	0.95	0.65–1.38
Leukemia	9567	144	0.93	0.79–1.10	23342	76	0.82	0.65–1.03
CUP^1^	9567	144	1.00	0.85–1.18	15566	61	0.98	0.76–1.26
All (including testis)	5521	4190	**1.12**	1.07–1.16	698512	3101	**1.09**	1.05–1.13

Bolding of RRs shows that 95%CI does not include 1.00. CUP^1^ = cancer of unknown primary.

FDRs = first degree relatives. RR = relative risk. CI = confidence interval.

In Table 3, results are shown for testicular seminoma. Seminoma was associated with the same cancers as all TC, and additionally with pancreatic (1.25) and endometrial cancers (1.25) and with Hodgkin lymphoma (1.48). The RR of 1.18 for association of seminoma with all cancer was higher than that for all TC. Only two specific associations were found in the reverse analysis; RR was 1.16 for prostate cancer and it was 1.28 for melanoma. When analyses were conducted by number of cases in family members (data not shown), risk of nervous system cancer was increased to 4.47 in two families with two patients diagnosed with seminoma (95% CI 1.12–17.88), and risk of non-Hodgkin lymphoma was increased to 5.34 in two families with two patients diagnosed with seminoma (95% CI 1.33–21.34).

**Table 3:** Risk of cancer in offspring when family members were diagnosed with testicular cancer.

Cancer site family member	Cases with negative family history	Relatives with discordant cancers			Cases with negative family history	Relatives with testicular cancers Seminoma		
		Any FDRs				Any FDRs		
		Cases	RR	95%CI		Cases	RR	95%CI
Upper aerodigestive tract	5081	52	0.87	0.66–1.14	14620	33	1.00	0.71–1.40
Stomach	5068	65	0.82	0.65–1.05	10645	17	0.70	0.44–1.13
Colorectum	4813	320	1.09	0.98–1.23	61114	139	0.98	0.83–1.15
Liver	5081	52	0.84	0.64–1.10	11399	24	0.94	0.63–1.40
Pancreas	5051	82	**1**.**25**	1.00–1.55	12440	37	1.28	0.93–1.77
Lung	4902	231	**1**.**15**	1.01–1.32	45805	106	1.02	0.84–1.24
Breast (female and male)	4713	420	**1**.**10**	1.00–1.22	117501	301	1.05	0.94–1.18
Cervix	5087	46	0.87	0.65–1.17	13379	28	0.90	0.62–1.30
Endometrium	5040	93	**1**.**25**	1.01–1.53	18113	40	0.92	0.68–1.26
Ovary	5066	67	1.12	0.88–1.43	15417	38	1.01	0.74–1.40
Prostate	4655	478	1.07	0.98–1.18	104535	291	**1**.**16**	1.03–1.30
Kidney	5057	76	0.99	0.79–1.24	16116	39	1.05	0.77–1.47
Urinary bladder	5002	131	1.04	0.87–1.24	24864	56	0.98	0.76–1.28
Melanoma	4966	167	**1**.**34**	1.15–1.57	41611	127	**1**.**28**	1.08–1.53
Skin	5025	108	0.91	0.75–1.10	21948	54	1.07	0.82–1.39
Nervous system	5005	128	**1**.**29**	1.08–1.54	32203	83	1.11	0.90–1.38
Thyroid glands	5100	33	1.24	0.88–1.75	8654	22	1.10	0.72–1.67
Endocrine glands	5085	48	0.90	0.68–1.20	14988	29	0.82	0.57–1.18
Connective tissue	5110	23	1.18	0.79–1.78	5558	13	1.02	0.59–1.75
Non-Hodgkin lymphoma	5041	92	1.03	0.84–1.27	23208	55	1.02	0.78–1.33
Hodgkin lymphoma	5108	25	**1**.**48**	1.00–2.19	6188	14	1.00	0.59–1.68
Myeloma	5098	35	0.96	0.69–1.34	7042	16	0.97	0.59–1.58
Leukemia	5048	85	1.00	0.81–1.25	23374	44	0.83	0.62–1.11
CUP^1^	5051	82	1.01	0.81–1.26	15590	37	1.03	0.75–1.42
All (including testis)	2762	2371	**1**.**18**	1.12–1.25	699821	1792	**1**.**09**	1.04–1.15

Bolding of RRs shows that 95%CI does not include 1.00. CUP^1^ = cancer of unknown primary.

FDRs = first degree relatives. RR = relative risk. CI = confidence interval.

Analyses were also carried out for nonseminoma (data not shown). RRs for nonseminoma were not increased with any discordant cancer. However, prostate cancer was increased to 1.15 when 201 sons were diagnosed with nonseminoma (1.00–1.32), and other male genital cancers were increased to 2.36 when six sons were diagnosed with nonseminoma (1.06–5.26). All cancers were increased to 1.07 when 1245 sons were diagnosed with nonseminoma (1.01–1.13).

## Discussion

TC has been associated with some other cancers as described in the introduction but the advantage of the present study is its size and its design in aiming at internal validation of the results in partially independent two-way analyses. We could show that all testicular cancers were associated with breast cancer and melanoma in both of the two-way analyses. Single significant associations were found with lung, ovarian, prostate and nervous system cancers. Most site-specific associations of TC were for seminoma, which was associated with melanoma in two ways, and with nervous system cancer in two different family types. Associations of seminoma with individual significant sites included pancreatic, lung, breast, endometrial and prostate cancers and Hodgkin and non-Hodgkin lymphomas. Site-specific associations of nonseminoma only included single significant results with prostate and other male genital cancers. Seminoma showed several discordant associations with other cancers, which may be related to an 8-year later onset compared to nonseminoma and underlying mechanistic differences.

Single discordant familial associations, listed above, may be of interest but they may also be chance findings and thus need to be confirmed in independent settings. Associations of TC with breast and nervous system cancers and melanoma were confirmed in at least two separate analyses and only the association with melanoma was reported previously^14,15^. It, however, appears noteworthy that, in addition to breast cancer, other hormone-dependent female sites were associated with TC in single analyses including ovarian and endometrial cancers, and prostate cancer was an associated male site. These are common cancers and the magnitude of relative risk was rather modest. It may, however, be premature to consider such a clustering of hormone-dependent cancers to be fortuitous.

Although concordant familial risks for TC are well-known, the present results contributed to the notion that nonseminoma appeared to be more genetically predisposed than seminoma because the risks were very high in families where more than two males were affected but case numbers were few^5,6,13^. Genome-wide association studies (GWAS) have identified some 50 single nucleotide polymorphisms (SNPs), which increased the risk of TC and many of these suggest disruption of developmental transcriptional regulation of germ cell differentiation as a basis of TC susceptibility^16,17^. It is, however, not well-known if SNPs may differentiate risks of seminoma and nonseminoma. The involved pathways cover KIT/KITLG/MAPK signalling, telomerase function, microtubule assembly and DNA damage repair^16^. The most prominent gene is KITLG that shows an allelic risk of 2.55 with a high population frequency of 0.8 for the risk allele^18^. The gene encodes the ligand for tyrosine-kinase KIT, which is commonly somatically mutated in seminomas. It has been calculated for 39 independent SNPs that these accounted for 37% of the (father-to-son) familial risk of TC^17^. The calculated polygenic risk score for men in the top 1% of genetic risk have a risk of 14, translating to a 7% lifetime risk of TC^16^. These data can be compared with our lifetime risk calculations in the Nordic familial setting^13^. The lifetime risk in the population was 0.6% and it was 2.3% for the brother of an affected individual, increasing to 10.3% for persons with multiple affected family members. As most of the present familial cases (2% of all TC) were affected brothers. Therefore, one can conclude that empirical familial risk and polygenic risk scores only partially overlap.

In summary, nonseminoma appeared to show higher familial associations with TC than seminoma but seminoma had many more familial associations with other cancer than nonseminoma. Associations of TC with breast and nervous system cancers and melanoma were internally consistent and mostly novel. Clustering of TC with hormone-dependent cancers of the breast, ovary, endometrium and prostate may have pathophysiological underpinnings, which could be related to interactions of endocrine disruptive chemicals or other unknown environmental factors with the host genetic background^6,8^.

## Subjects and Methods

In the Swedish “Multigeneration Register” individuals who were born in Sweden in 1932 and onwards are registered with their parents. We define these as the offspring and parental generation, respectively^19^. The Swedish “Multigeneration Register” was linked by the individually unique national registration number to the Cancer Registry for the years 1958–2015 to make the basis of the Family-Cancer Database. In the Database families are organized in subsequent generations. The Swedish personal number is issued to all permanent residents upon birth or immigration to Sweden. The completeness of the cancer registry is considered to be over 90%^20^. The cancer site is registered using diagnostic codes according to the 7th (and later) revisions of the International Classification of Diseases (ICD-7). Patients diagnosed with TC were identified as were all other cancers in family members. The histological classification of TCs was used, as present in the Cancer Registry, to define seminoma and non-seminoma.

Relative risks (RRs) were used to measure cancer risks for TC in the offspring generations according to occurrence of cancers in their first-degree family members (parents, siblings or children). The parental generation born before 1932 was diagnosed with 1427 TC and served as probands only and were not used for person-year calculations. In one family TCs were diagnosed in three generations, and the person in the middle generation was entered separately as a case and a proband. In the reverse analysis, RR was calculated for cancer in offspring when family members were diagnosed with TC. These two types of analyses were partially independent, particularly for discordant cancers, and positive results in both analyses provided strong support for a true association. Follow-up was started for each offspring at birth, immigration or January 1^st^, 1958, whichever came latest. Follow-up was terminated on diagnosis of first cancer, death, emigration, or the closing date of the study, which was December 31^st^, 2015. Parents’ ages were not limited but sons were 0 to 83 years of age; siblings could be defined only in the offspring generation.

Poisson regression modeling was employed to estimate RRs and corresponding 95% confidence intervals (CI). Potential confounders, including sex, age group (5-year bands), period (5-year bands), socioeconomic status (blue-collar worker, white-collar worker, farmer, private, professional, or other/unspecified), residential area (large cities, South Sweden, North Sweden, or unspecified) were added to the model as covariates. SAS version 9.4 was used to perform the statistical analysis.

### Ethical statement

The study was approved by the Ethical Committee of Lund University and the study was conducted in accordance with the approved guidelines not requesting informed consent. The study is national register-based study on anonymous personal data.
